# A Spectrometric Method for Hydrogen Peroxide Concentration Measurement with a Reusable and Cost-Efficient Sensor

**DOI:** 10.3390/s151025716

**Published:** 2015-10-12

**Authors:** Cheng-Chih Hsu, Yuan-Rong Lo, Yu-Chian Lin, Yi-Cen Shi, Pang-Lung Li

**Affiliations:** Department of Photonics Engineering, Yuan Ze University, 135 Yuan-Tung Road, Chung-Li 32003, Taiwan; E-Mails: s1010756@mail.yzu.edu.tw (Y.-R.L.); s1020759@mail.yzu.edu.tw (Y.-C.L.); s1020729@mail.yzu.edu.tw (Y.-C.S.); s1010716@mail.yzu.edu.tw (P.-L.L.)

**Keywords:** optical sensor, spectrometry, hydrogen peroxide

## Abstract

In this study we developed a low cost sensor for measuring the concentration of hydrogen peroxide (H_2_O_2_) in liquids utilizing a spectrometric method. The sensor was tested using various concentrations of a peroxidase enzyme immobilized on a glass substrate. H_2_O_2_ can be catalyzed by peroxidase and converted into water and oxygen. The reagent 4-amino-phenazone takes up oxygen together with phenol to form a colored product that has absorption peaks at 510 nm and 450 nm. The transmission intensity is strongly related to the hydrogen peroxide concentration, so can be used for quantitative analysis. The measurement range for hydrogen peroxide is from 5 × 10^−^^5^% to 1 × 10^−3^% (0.5 ppm to 10 ppm) and the results show high linearity. This device can achieve a sensitivity and resolution of 41,400 (photon count/%) and 3.49 × 10^−5^% (0.35 ppm), respectively. The response time of the sensor is less than 3 min and the sensor can be reused for 10 applications with similar performance.

## 1. Introduction

Hydrogen peroxide (H_2_O_2_) has many applications [1,2,3,4], including as a bleaching agent for everything from teeth to wood pulp, for the treatment of wastewater and effluent, encouraging the root growth of rice seedlings, and as a disinfectant in the food and pharmaceuticals industry. To measure the H_2_O_2_ concentration, several methods [5,6,7,8,9,10,11] including optical interferometry, spectrophotometric, fluorimetric, and chemiluminescence methods have been proposed. Chiu *et al.* [5] and Chen *et al.* [6] demonstrated the use of a heterodyne interferometer to measure various concentrations of solution with high sensitivity (3 × 10^−5^ RIU) and resolution (0.06%) within a wide measurement range of solution concentrations. However, their method could not determine the concentration of specific chemical components within a complicated mixture. Chen *et al.* [7] fabricated a H_2_O_2_ sensor with entrapped horseradish peroxidase by using mesoporous silica deposited on a polyaniline modified platinum electrode. Their results showed a good linearity of response between the cathode and H_2_O_2_ concentration within the range of 0.02 to 18.5 mM. In addition, their proposed sensor preserved 80% of the enzymatic activity after 16 days. Tanner *et al.* [8] proposed a novel OPDV UV absorption method for measuring the H_2_O_2_ concentration in rainwater. The major advantages of the OPDV method are the high stability of the reagent and low interference effects between the reagent and the inorganic constituents in the rainwater. They obtained a detection limit of 5.8 nmol for 20 cm^3^ rainwater. Vieira and Fatibello-Filho [9] developed an enzymatic source of peroxidase by extraction from zucchini. In the guaiacol, H_2_O_2_, and peroxidase reaction, strong absorbance could be measured at 470 nm by a spectrophotometric flow system. They evaluated the concentration of guaiacol obtained with their proposed method and showed a low detection limit of 2.1 × 10^−6^ mol/L at a guaiacol concentration of 0.05 mol/L. El-Essi *et al.* [10] developed an H_2_O_2_ sensor that used the sol-gel method for determining the H_2_O_2_ concentration. They monitored the absorbance of oxidized variamine blue at a wavelength of 550 nm and evaluated the performance of the proposed sensor under various conditions of pH, concentration, temperature and stability of variamine blue. Onoda *et al.* [11] developed a phosphine-based fluorescent reagent to determine the H_2_O_2_ concentration with fluorometric analysis. Their method provided a rapid derivatization reaction within 2 min at room temperature. Rapoport *et al.* [12] used a special assay that included superoxide dismutase, catalase, and methanol in the tested reaction system where the H_2_O_2_ concentration can be obtained by analyzing the fluorescence signal. Feng *et al.* [13] reported on a KMnO4-OP chemiluminescence method. They demonstrated the influence of the acid selection, potassium permanganate concentration, and sensitizer selection on the proposed method. Regardless of the type of measurement technique applied, all these proposed methods employ a complex chemical reaction to form an indicator, which produces absorbance variations, or fluorescent emissions, or acts as an illuminator. Analysis of the variations in the absorbance, fluorescence intensity, or illumination at a specific wavelength is needed to obtain the H_2_O_2_ concentration. Furthermore, none of these methods can be used to provide a reusable H_2_O_2_ sensor, the consequence of which is the consumption of vast quantities of reacted chemicals for measuring the H_2_O_2_ concentration. The comparisons of the proposed methods are summarized in Table 1.

To reduce the cost of the reacted chemicals, we fabricated an enzymatic H_2_O_2_ sensor by immobilizing peroxidase enzyme (POD) on a glass substrate with various POD concentrations. The fabrication procedure for production of the proposed sensor is simple and reproducible. Based on the chemical reaction of the proposed method, the primary absorption peak is at 510 nm. The transmission intensity at a wavelength of 510 nm is strongly related to the H_2_O_2_ concentration so can be used for quantitative analysis. The results show high linearity within a range of H_2_O_2_ concentrations from 5 × 10^−^^5^% to 1 × 10^−3^%. The sensitivity and resolution can be as high as 41,400 (photon count/%) and 3.49 × 10^−5^%, respectively. Furthermore, the proposed sensor exhibits a shorter response time (less than 3 min) than other methods and offers reproducible performance over 10 applications.

**Table 1:** Comparisons of the proposed methods.

Ref.	Method	Enzyme	Sensor Property
[5,6]	Interferometry	None	D-type fiber/right angle prism
[7]	Amperometric	Horseradish peroxidase (HRP)	SBA-15 entrapped HRP deposited on polyaniline
[8]	Spectrometric	None	No rigid fabricated sensor, solutions of pyridine-2 6-dicarboxylic acid, H_2_O_2_, and vanadate
[9]	Spectrometric	Peroxidase from zucchini	No rigid fabricated sensor, reaction with solutions of supernatant, guaiacol, H_2_O_2_, and peroxidase
[10]	Spectrometric	Horseradish peroxidase (HRP)	HRP entrapped in silicate glass matrix with Sol-Gel method
[11]	Fluorimetric	None	No rigid fabricated sensor, reaction with solutions of phosphine-based fluorescent reagent, H_2_O_2_, and sodium tungstate dihydrate
[12]	Fluorimetric	Superoxide dismutase (SOD)	No rigid fabricated sensor, reaction with solutions of SOD, H_2_O_2_, and Nash reagent
[13]	Chemiluminescence	None	No rigid fabricated sensor, reaction with solutions of octylphenyl polyglycol ether (OP), acidic KMnO_4_, and H_2_O_2_
This work	Spectrometric	Horseradish peroxidase (HRP)	HRP immobilized on glass substrate

## 2. Methods and Materials

### 2.1. Chemicals and Sensor Preparation

All chemicals used in the experiments were purchased from commercial sources. A 35% (v/v) hydrogen peroxide solution (Nihon Shiyaku Industries, Kyoto, Japan) was used to prepare a set of nine solutions (from 1 × 10^−4^% (v/v) to 1 × 10^−3^% (v/v)) which were diluted with distilled water (DI water). The POD (EC 1.11.1.7, from horseradish peroxidase, 150–250 units/mg), 4-aminoantipyrine (EC 201-452-3), 3-aminopropyltriethoxysilane (EC 213-048-4), 3-sulfo-N-hydroxysuccinimide ester (B1022) and phenol (EC 203-632-7) were obtained from Sigma-Aldrich (St. Louis, MO, USA).

**Figure 1:**
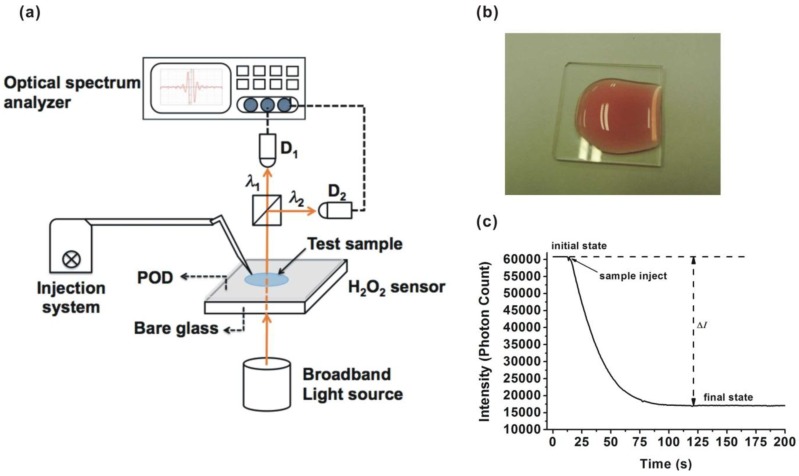
Measurement system and H_2_O_2_ sensor: (**a**) system arrangement; (**b**) photograph of the H_2_O_2_ sensor; and (**c**) transmittance-time response curve.

The H_2_O_2_ sensor was fabricated by immobilizing the POD on bare glass. The surface of the bare glass (dimensions: 2.5 cm × 2.5 cm) was modified by treatment with 5% (v/v) 3-aminopropyltriethoxysilane (APTES) in ethanol for 10 min at room temperature and then heated at 120 °C for 30 min. After that, 500 μL of 0.005% suberic acid bis (3-sulfo-N-hydroxysuccinimide ester) sodium salt (BS3) mixed with 10 mM/L phosphate buffered saline (PBS) covered the glass at room temperature for 20 min and was then removed. The pH of PBS was controlled at 7. This procedure was performed to modify the glass surface for easy immobilization of the POD. Subsequently, the modified surfaces were covered with different POD solutions with concentrations of 0.001 mg/mL, 0.0005 mg/mL, and 0.0003 mg/mL at pH 7 and 1 h. The unreacted aldehyde groups were then quenched with a 15 mM Tris buffer solution at room temperature for 10 min. The hydrogen peroxide sensor with the result of chromogen testing is shown in Figure 1b. The fabricated H_2_O_2_ sensor was stored in a refrigerator at a temperature of 4 °C.

### 2.2. Method and Apparatus

A schematic diagram outlining the proposed method is shown in Figure 1a. The visible transmission spectra were recorded using an optical spectrum analyzer (model: SD1200-LS-HA, OTO Photonics Inc., Hsinchu, Taiwan) with a spectral resolution of 1.3 nm. The broadband light source used was passed through the H_2_O_2_ sensor, and then divided into two paths by a beam splitter. These two beams were detected by photodetectors D_1_ and D_2_, which monitored two different wavelengths λ_1_ and λ_2_. The optical spectrum analyzer recorded the variation in the transmission spectra at λ_1_ and λ_2_ as the chemical reaction progressed. Transmittance-time response experiments were conducted to monitor changes in the transmittance (photon count variation) before and after injecting a mixed solution containing a chromogen reagent and H_2_O_2_ solution. As the reaction progressed, the concentration of the colored product increased and the transmission intensity decreased. The chemical reaction was terminated when the concentration of the colored product no longer increased and the transmission intensity remained unchanged at the final state. Figure 1c shows the transmittance-time response of the proposed sensor and $\Delta I=\left|PC_{\text{initial}}-PC_{\text{final}}\right|$ which is related to the H_2_O_2_ concentration, where *PC*_initial_ and *PC*_final_ are the photon count at the initial state and final state, respectively.

The measurement procedure is described below. The chromogen reagent was prepared with 6.4 mg of 4-aminoantipyrine and 42 mg of phenol, which were dissolved in 40 mL of a PBS solution. The mixed solution included 8 mL of a chromogen reagent and 2 mL of the test sample (H_2_O_2_ solution in various concentrations). After the mixed solution is injected, the H_2_O_2_ will be converted to water and oxygen by POD. An oxygen acceptor 4-aminophenazone takes up the oxygen and together with phenol forms a colored product. The chemical reaction can be written as [14,15]:

(1) $$2{\text{H}}_{\text{2}}{\text{O}}_{\text{2}}+\text{phenol}+\text{4}-\text{aminophenazone}\overset{\text{POD}}{\to }{\text{4H}}_{\text{2}}\text{O}+\text{colored product}$$

For the reliability testing of the proposed sensor, repeated applications of H_2_O_2_ solution with a concentration of 0.001% were performed. Every 10 applications, the chromogen test was adopted to evaluate the activity of POD.

## 3. Experimental Results and Discussion

**Figure 2:**
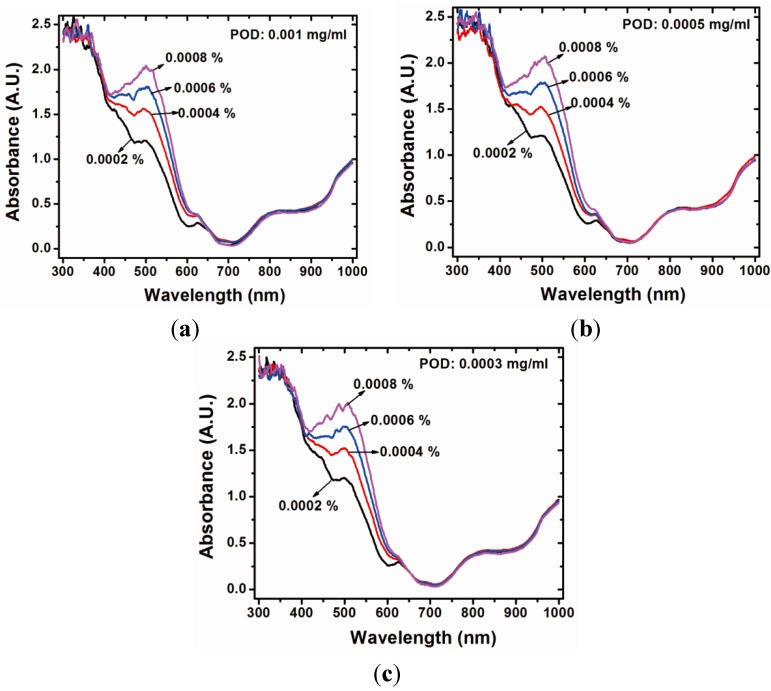
Absorbance behavior of the proposed sensor with various POD concentrations: (**a**) 0.001 mg/mL; (**b**) 0.0005 mg/mL; and (**c**) 0.0003 mg/mL.

The absorbance of the colored product under various hydrogen peroxide concentrations is shown in Figure 2. An examination of Figure 2a shows that the absorbance of the colored product is strongly related to the H_2_O_2_ concentration within the wavelength range of 350 nm to 600 nm. Obviously, the optimal variation of absorbance is monitored at 510 nm and 450 nm for comparison in this study. In addition, Figure 2 shows the similarity of behavior of the absorbance under various concentrations of POD.

To demonstrate the performance of the proposed sensor, various concentrations of H_2_O_2_ were prepared for measurement. In this study, the H_2_O_2_ concentration was within the range of 10^−3^%−10^−4^%, the POD concentration was controlled at 0.001, 0.0005 and 0.0003 mg/mL and the volumes of the chromogen solution and H_2_O_2_ were 8 mL and 2 mL, respectively. The wavelength was monitored at 510 nm and 450 nm.

**Figure 3:**
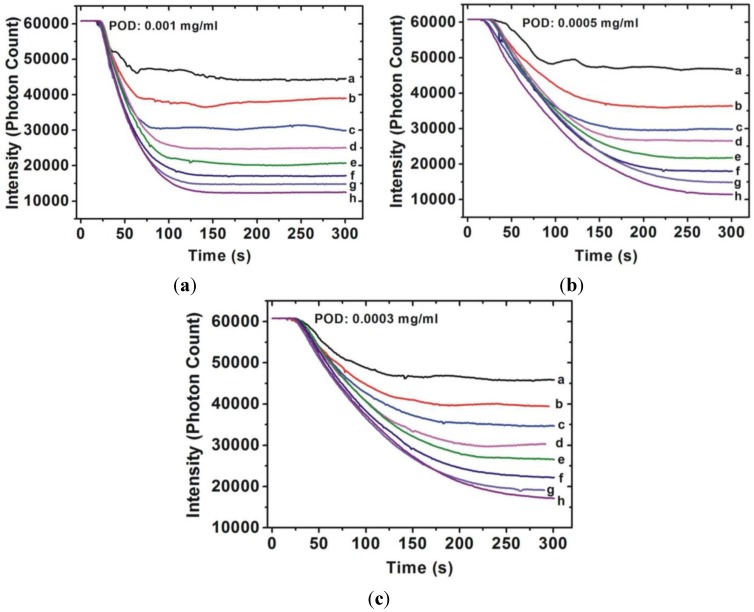
Transmittance-time response behavior of various H_2_O_2_ concentrations (a: 0.0001%; b: 0.00015%; c: 0.0002%; d: 0.00025; e: 0.0003%; f: 0.00035; g: 0.0004; h: 0.00045%) measured by the sensor with various POD concentrations ((**a**) 0.001 mg/mL; (**b**) 0.0005 mg/mL; and (**c**) 0.0003 mg/mL) at monitored wavelengths of 510 nm.

**Figure 4:**
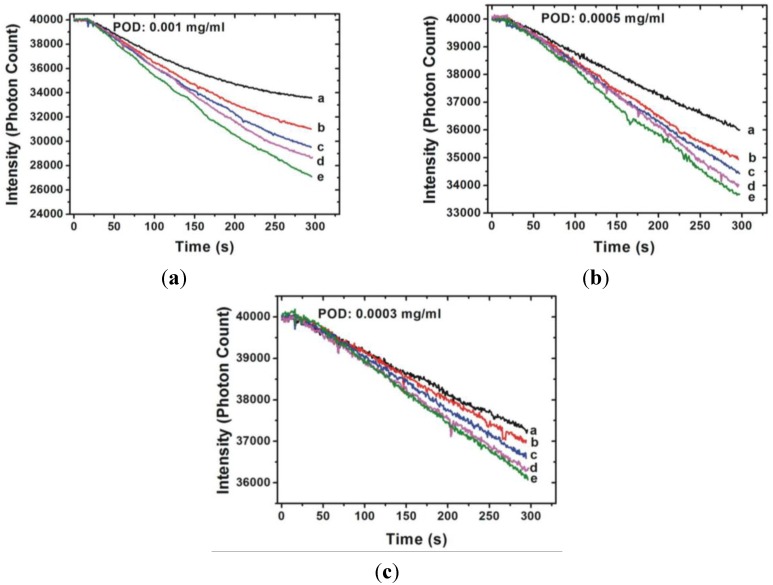
Transmittance-time response behavior of various H_2_O_2_ concentrations (a: 0.0001%; b: 0.00015%; c: 0.0002%; d: 0.00025; e: 0.0003%) measured by the sensor with various POD concentrations ((**a**) 0.001 mg/mL; (**b**) 0.0005 mg/mL; and (**c**) 0.0003 mg/mL) at monitored wavelengths of 450 nm.

The transmittance-time response curves of various hydrogen peroxide concentrations are shown in Figure 3 and Figure 4. Figure 3 shows the results obtained when the monitoring wavelength was at 510 nm and Figure 4 shows the results of monitoring at 450 nm. As can be seen in Figure 3, the reaction time is strongly related to the POD concentration, indicating that the higher the POD concentration, the shorter the reaction time. The reaction time was shorter than 200 s when the POD concentration was 0.001 mg/mL. In contrast to the results obtained when the monitoring wavelength was at 510 nm, the reaction time cannot easily by judge when the monitored wavelength is 450 nm. No matter what wavelength is considered for monitoring, Δ*I* is related to the hydrogen peroxide concentration. In other words, Δ*I* depends on the absorption behavior of the colored product.

Figure 2 shows stronger absorption of the colored product at the wavelength of 510 nm. The more material that is absorbed, the greater the change of the photon count of the transmission light (Δ*I*). The Δ*I* of each hydrogen peroxide concentration was smaller when the monitoring wavelength was 450 nm than the results obtained when monitoring at 510 nm. Furthermore, it is difficult to determine the termination of the chemical reaction when the monitoring wavelength is 450 nm. The reason might be because the intermediate product is still generated. By contrast with the results with a monitoring wavelength of 510 nm, the termination of the reaction is easy to determine and is within approximately 300 s.

**Figure 5:**
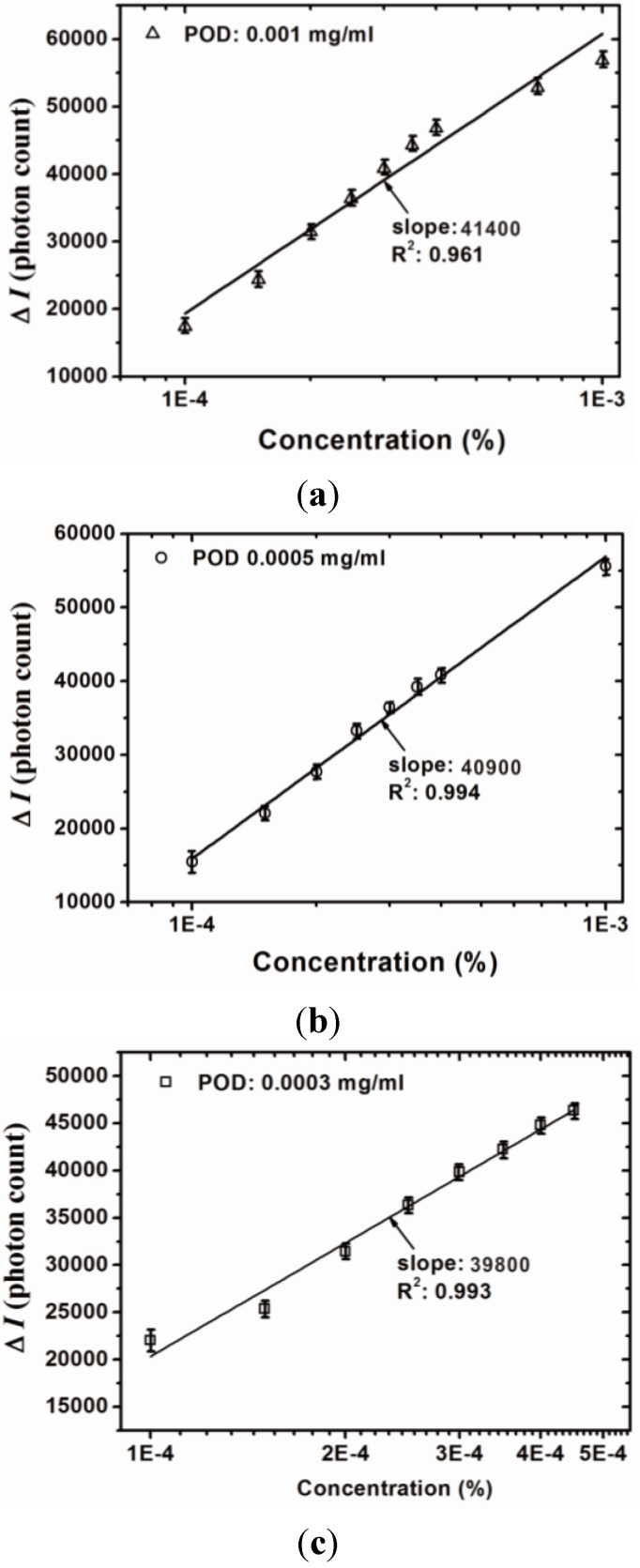
Calibration curve of the proposed sensor with various POD concentrations ((**a**) 0.001 mg/mL; (**b**) 0.0005 mg/mL; and (**c**) 0.0003 mg/mL) at a monitored wavelength of 510 nm.

**Figure 6:**
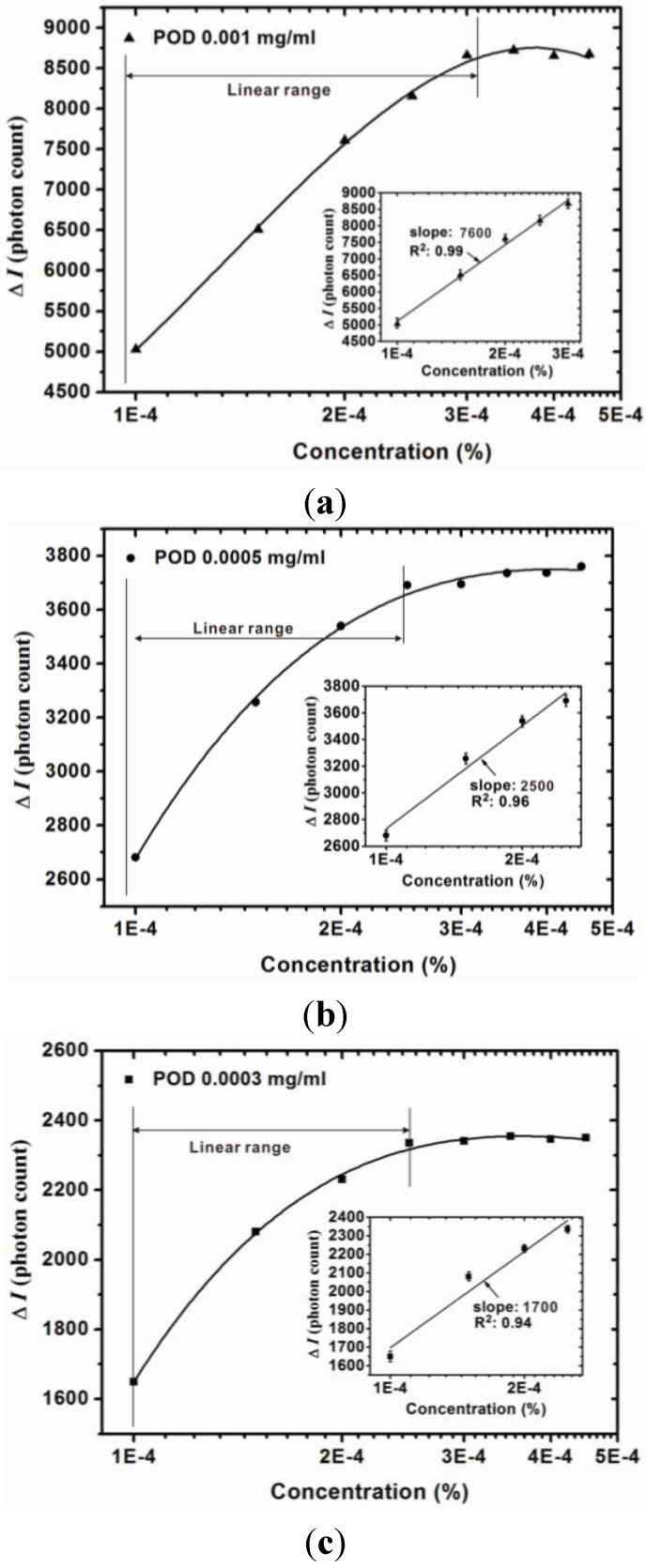
Calibration curve of proposed sensor with various POD concentrations ((**a**) 0.001 mg/mL; (**b**) 0.0005 mg/mL; and (**c**) 0.0003 mg/mL) at a monitored wavelength of 450 nm.

The quantitative analysis results of the proposed sensor are shown in Figure 5 and Figure 6, which indicate the calibration curve measured by using the proposed sensor with different POD concentrations and monitored wavelengths. Measurements were performed 10 times and the mean values and standard deviation were plotted. It is clear that the measurement range of the H_2_O_2_ concentration is related to the monitoring wavelength and exhibits greater linearity when the monitoring wavelength is 510 nm. The slope of the calibration curve indicates the sensitivity of the proposed sensor. It can be seen that the sensitivity increases with an increasing POD concentration. In addition, the sensitivity is also related to the monitoring wavelength and the results show that greater sensitivity can be obtained when the monitoring wavelength is 510 nm. In contrast, when monitoring at 450 nm, the calibration curve exhibited saturation as H_2_O_2_ concentration reached 3 × 10^−4^%. The linear ranges and corresponding calibration curves of the proposed sensors monitored at a wavelength of 450 nm are shown in Figure 6. The optimal measurement conditions are obtained when the POD concentration is controlled at 0.001 mg/mL and the monitoring wavelength is 510 nm. The resolution of the proposed sensor can be represented as [16]:

(2) $$\Delta I_{\text{res}}=K+S\cdot \log\left[\Delta C\right]$$

where *K* and *S* are the intersection and slope of the calibration curve, respectively; Δ*I*_res_ and Δ*C* represent the resolutions of the photon counter and concentration, respectively.

**Figure 7:**
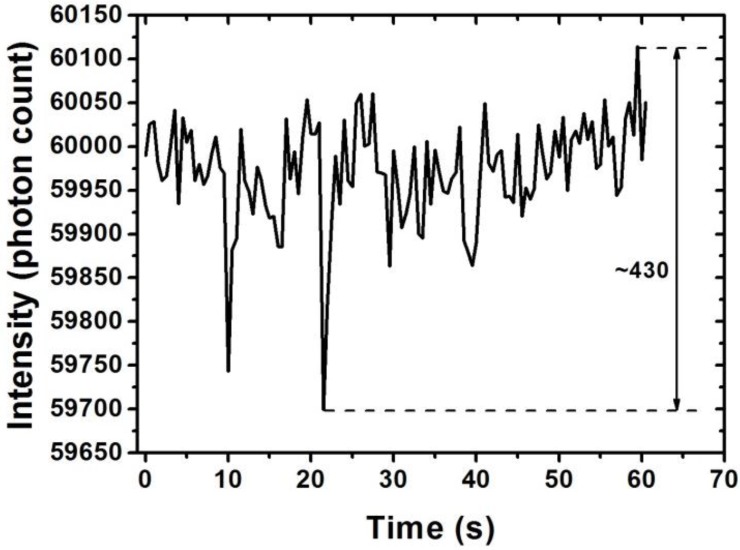
Stability evaluation of the photon counter.

It is obvious that Δ*I*_res_, which is the result of the photon counter resolution (theoretically one photon can be detected, but unexpected electronic noise will decrease the accuracy of photon counting), will affect the resolution of the proposed method. Therefore, the actual Δ*I*_res_ can be indicated by stability evaluation of the photon counter, as shown in Figure 7. Stability evaluation of the proposed sensor was conducted with a bare H_2_O_2_ sensor by recording the transmission intensity variation at the monitored wavelength of 510 nm within 1 min. The largest intensity variation was approximately 430 photon counts.

The resolution of the proposed method can be calculated using Equation (2) and the results are summarized in Table 2. The results show that the sensitivity of the proposed sensor decreases as the POD concentration increases. With a high POD concentration the proposed sensor exhibits high sensitivity regardless of whether the monitoring wavelength is at 510 nm or 450 nm. Furthermore, the resolution of the proposed sensor decreases as the POD concentration decreases, the optimal resolution being obtained with a high POD concentration. Therefore, the optimal fabrication condition for the proposed sensor is with a POD concentration of 0.001 mg/mL and the optimal sensitivity and resolution are 41,400 (photon count/%) and 3.49 × 10^−5^%, respectively.

**Table 2:** Sensitivity and resolution of the proposed sensor.

Monitoring Wavelength (nm)	POD Concentration (mg/mL)	Sensitivity (Photon Count/%)	Δ*I*_res_	Resolution	
				Theoretical (10^−8^%)	Real (10^−5^%)
510	0.001	41,400		8.11	3.49
	0.0005	40,900	430	9.72	4.18
	0.0003	39,800		11.74	5.05
450	0.001	7600		10.37	4.46
	0.0005	2500	430	12.37	5.32
	0.0003	1700		16.23	6.98

**Figure 8:**
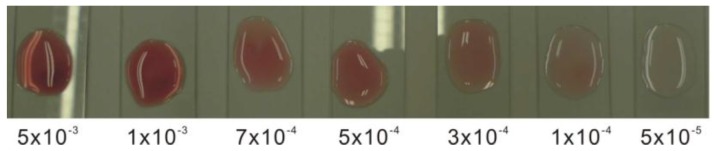
Qualitative analysis of the proposed sensor. The H_2_O_2_ concentration varied from 5 × 10^−5^% to 5 × 10^−^^3^% with the POD concentration controlled at 0.001 mg/mL.

Figure 8 illustrates the results of qualitative analysis obtained by using the proposed sensor (POD concentration: 0.001 mg/mL) to measure various H_2_O_2_ concentrations. In the color chart comparison method [15], the approximate quantity of an unknown concentration can be obtained by comparison with an indicator color chart. It is obvious that the colored product generated by the proposed sensor gets darker in color as the H_2_O_2_ concentration increases and can be easily distinguished visually. The detection limit of qualitative analysis is approximately 5 × 10^−5^%.

**Figure 9:**
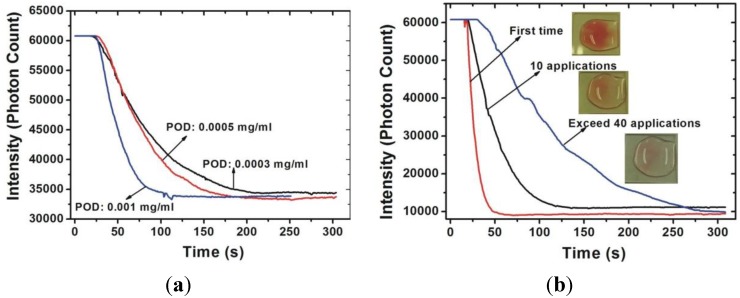
Reliability of the proposed sensor: (**a**) transmittance-time response curves with various POD concentrations; and (**b**) transmittance-time response curves for various application numbers measured by the proposed sensor with POD concentration of 0.001 mg/mL.

Figure 9 demonstrates the reliability of the proposed sensor as indicated by the transmittance-time response curves for two conditions. First, the POD concentration will affect the chemical reaction of catalytic POD. A higher POD concentration leads to a faster reaction which causes a shorter response time and sharper tendency of the transmittance-time response curve. Figure 9a shows the transmittance-time response curves for the test samples measured by the proposed sensor with various POD concentrations. It is clear that the reaction time decreases as the POD concentration is increased and the optimal reaction time is shorter than 150 s as measured by the sensor with a POD concentration of 0.001 mg/mL. In addition, the tendency of the transmittance-time response curve is sharper with a higher POD concentration. Second, the activity of the POD will decrease as the number of applications of the proposed sensor increase and the reaction time of the proposed sensor will increase as the application number increases. Moreover, the color of the colored product will become lighter as the number of applications increases. As can be seen in Figure 9b, the behavior of the transmittance-time response of the proposed sensor remains identical for 10 applications. When the application of the sensor exceeds 40 times, the response time of the chemical reaction will exceed 300 s. Combined with the chromogen results, shown in the inset of Figure 9b, it can be seen that the activity of the POD immobilized on the proposed sensor is still operative but the efficiency in converting the H_2_O_2_ into oxygen and water is lower. Based on these results, we can conclude that the proposed sensor can preserve similar behavior (chemical reaction terminated within 150 s) over 10 applications. Furthermore, it can still be used even after 40 applications, in which case the chemical reaction will terminate within 360 s.

The performance of the proposed sensor was compared with the performance of methods described in some other previous works and noted in Introduction section. The results of the comparison are summarized in Table 3 mentioning key aspects such as the detection limit, resolution, linear range, response time, and reusability. As shown by the comparison, the proposed method provided a reusable H_2_O_2_ sensor with high sensitivity, low detection limit, and fast determination of H_2_O_2_ concentration within the measurement range of 10^−4^%–10^−3^%. Furthermore, the proposed sensor has both quantitative and qualitative characteristics. Therefore, the proposed sensor can be adopted for visual detection in food-safety evaluation or determination the residual H_2_O_2_ concentration released from a single-use chopstick.

**Table 3:** Performance of different H_2_O_2_ concentration measurement methods.

Ref.	Detection Limit	Resolution	Linear Range	Response Time	Reusability
[5,6]	10%	0.06%	10%–90%	X	X
[7]	0.02 mM	417.5 μA/mM	0.04–12 mM	~5 s	X
[8]	5.8 nmol	X	0.05–50 ppm	X	X
[9]	2.1 × 10^−^^6^ mol/L	X	16–660 μmol/L	X	X
[10]	5 × 10^−5^ M	X	Nonlinear	1 h	X
[11]	1.25 ng/μL	X	1.25–50 ng/μL	<2 min	X
[12]	5 μM	X	Nonlinear	X	X
[13]	0.006 μmol/L	X	10^−8^–6 × 10^−5^ mol/L	X	X
This work	3.5 × 10^−5^%	5 × 10^−5^%	10^−4^–10^−3^%	<5 min	10 times

## 4. Conclusions

We fabricated a cost-efficient H_2_O_2_ sensor which uses immobilized POD on bare glass with a simple and reproducible fabrication procedure. By using transmission spectrum analysis and the colorimetric method, we demonstrated the quantitative and qualitative characteristics of the proposed sensor. The proposed sensor can be applied as a portable sensor providing visual detection or an alternative precision quantitative instrument in the laboratory. The detection limits of qualitative and quantitative analysis were 5 × 10^−5^% (0.5 ppm) and 3.49 × 10^−5^% (0.35 ppm) as measured by the sensor with a POD concentration of 0.001 mg/mL. We also evaluated the sensitivity and resolution of the proposed sensor under various monitored wavelengths and POD concentrations. The results show that the measurement range of the H_2_O_2_ concentration, sensitivity and resolution of the proposed sensor increased as the POD concentration increased regardless of whether the monitoring wavelength was 510 nm or 450 nm. Moreover, when POD concentration was 0.001 mg/mL, the chemical reaction time was shorter and similar performance was preserved for 10 applications.
